# Salvianolic acid B alleviates depression-like behaviors by reducing neuronal injury and promoting neurogenesis in a manner associated with JAK-STAT signaling pathway inhibition

**DOI:** 10.3389/fneur.2026.1817261

**Published:** 2026-06-03

**Authors:** Ni Wang, Wei Guo, Xiaochen Yu, Yaqing Sun, Shujing Shi, Yan Xie

**Affiliations:** 1 Health Management Center of Liaoning Electric Power Central Hospital, Shenyang, Liaoning, China; 2 Health Department of Liaoning Electric Power Central Hospital, Shenyang, Liaoning, China

**Keywords:** depression, hippocampal neurons, JAK-STAT signaling pathway, neurogenesis, salvianolic acid B

## Abstract

**Background:**

Depression is strongly associated with hippocampal neuroinflammation, neuronal damage and neurogenesis. Salvianolic acid B (SalB) has anti-inflammatory and neuroprotective potential, but whether its antidepressant effect is achieved by regulating the JAK-STAT signaling pathway has not been systematically studied.

**Methods:**

HT22 cell injury was induced by corticosterone (CORT) and treated with SalB. The appropriate intervention dose was screened by CCK-8 assay. The degree of cell damage was detected by flow cytometry, ELISA and kit. A chronic unpredictable mild stress (CUMS) model of depression was induced, and behavioral tests were performed. Neuronal damage, microglia activation, neuronal apoptosis, neurogenesis and synaptic plasticity were detected by ELISA, immunofluorescence, immunohistochemistry, Nissl staining and Golgi-Cox staining. The activity of JAK-STAT pathway in hippocampus and cells was detected.

**Results:**

HT22 cells were exposed to 10, 15, and 20 μM SalB and 200 μM CORT, respectively. SalB inhibited CORT-induced release of inflammatory factors, oxidative stress and apoptosis. In CUMS mice, SalB significantly improved depression-like behavior, inhibited excessive activation of microglia, reduced neuroinflammation and hippocampal neuronal apoptosis, protected Nissl body structure and mature neurons, promoted neurogenesis and improved dendritic spine density and synaptic protein expression. In addition, SalB also inhibited JAK-STAT signaling, and pathway activator RO8191 reversed the neuroprotective effect of SalB.

**Conclusion:**

SalB exerted multiple protective effects against neuroinflammation, neuronal apoptosis and neurogenesis in a manner associated with inhibition of the JAK-STAT signaling pathway, and ultimately improved depression-like behavior.

## Graphical Abstract

**Figure:**
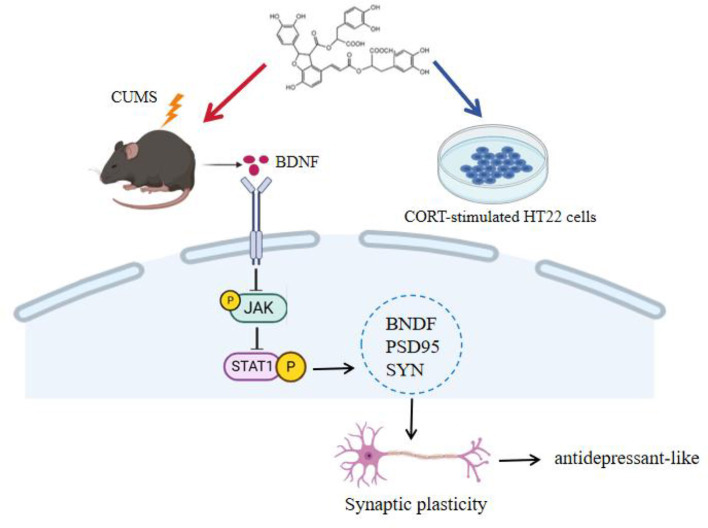
SalB reduced hippocampal neuronal damage and promoted neurogenesis, and these effects were associated with inhibition of JAK–STAT signaling pathway activation, consequently alleviated depression-like behaviors in CUMS mice.

## Highlights

- Salvianolic acid B (SalB) attenuated CORT-induced HT22 cell injury, and this effect was associated with reduced JAK-STAT signaling pathway.
- SalB alleviated depression-like behaviors and improved hippocampal neuron injury.
- SalB reduced the inflammatory response of hippocampal neurons and the activation of microglia in CUMS mice.
- SalB promoted neurogenesis in CUMS mice.

## 1 Introduction

Depression is an affective disorder commonly triggered by an interaction of multiple factors. Its core clinical features are persistent depression, anhedonia and cognitive impairment. The disease has the characteristics of high incidence and poor recovery rates (1, 2). The current clinical treatment mainly relies on chemical synthetic antidepressants, including selective serotonin-selective reuAG490ke inhibitors (SSRIs) and norepinephrine-dopamine reuptake inhibitors (NDRIs). However, these drugs have problems such as limited efficacy, slow onset, obvious side effects, and easy to produce treatment resistance, which severely affect patients' quality of living (3–5). Hence, exploring new targets and antidepressants is urgently needed to achieve faster onset, more lasting efficacy and lower side effects.

The pathogenesis of depression is complex, involving multi-system and multi-level pathophysiological changes. In mechanism of depression, it mainly includes neurotransmitter disorders, hippocampal neurogenesis disorders, impaired synaptic plasticity and neuroinflammation (6). Among them, the hippocampus, as a key hub of the limbic system, is central for emotional regulation, cognition and stress response. Its structural and functional impairment is regarded as the core pathological feature of depression. Anatomically, the hippocampus is mainly composed of two complex sub-structures: the dentate gyrus (DG) and the cornu ammonis (CA) area (7). It has obvious neuroplasticity and neurogenesis (8), which is the basis of various hippocampal-dependent learning and memory formation. Continuing stress can cause neuronal atrophy, including synaptic loss and hippocampal volume reduction (9). The neuroinflammation is a vital mechanism of depression (10), which has been proved to be a key bridge between stress stimulation and depression-like behavior. Microglia are immune cells that mediate synaptic pruning to maintain normal synaptic transmission during brain development (11). However, under pathological conditions, microglial cells are overactivated and release large amounts of inflammatory factors, which can directly damage neurons, inhibit hippocampal neurogenesis and lead to synaptic plasticity damage, and ultimately induce depression (12). At the same time, stress-induced neuronal apoptosis and dendritic atrophy further destroyed the integrity of the hippocampal neural circuit (13, 14). Therefore, neurogenesis disorders, impaired synaptic plasticity, and neuroinflammation are all causes of hippocampal dysfunction.

The Janus kinase/signal transducer and activator of transcription (JAK-STAT) signaling participates in immune inflammatory responses in different pathological processes and is vital for many diseases (15). This pathway is abnormally activated in patients with depression and animal models (16, 17). This pathway mediates pro-inflammatory cytokines production and microglial proliferation, leading to hippocampal synaptic defects in the depression model (18). Therefore, targeted inhibition of JAK-STAT pathway is expected to become promising new antidepressant strategies. In recent years, more and more evidence has shown that abnormal activation of this pathway is not only involved in the pathogenesis of depression, but also closely related to the pathological processes of various neurodegenerative diseases and neuroinflammation-related diseases (19). In Alzheimer's disease and Parkinson's disease, the continuous activation of JAK2/STAT3 signaling pathway can promote the production of pro-inflammatory cytokines, aggravate microglia-mediated neuroinflammatory response, and lead to neuronal apoptosis and synaptic dysfunction (20). Studies have shown that inhibition of the JAK2/STAT3 pathway can effectively reduce neuroinflammation and improve neuronal survival (21). In the model of cerebral ischemia-reperfusion injury, the activation of JAK-STAT pathway can regulate the M1/M2 polarization of microglia and affect the outcome of neuroinflammation and the recovery of neurological function (22). In summary, the JAK-STAT signaling pathway is a key molecular hub connecting neuroinflammation and neuronal damage in various central nervous system diseases. Intervention strategies targeting this pathway have broad application prospects in the field of neuroprotection.

Natural drugs have attracted significant focus in depression research because of their advantages of multi-target mechanism and low toxic and side effects. Salvianolic acid B (SalB) is an effective active ingredient of *Salvia miltiorrhiza* Bunge. It has low biological toxicity and is rich in phenolic hydroxyl groups. It is widely recognized for its potent antioxidant, anti-inflammatory and cardiovascular protective activities (23). In recent years, it has gradually gained widespread attention in nerve injury diseases. Studies have shown that Salvianolic acid exerts a neuroprotective function on dopaminergic neurons and inhibits the production of inflammatory mediators by microglia, thereby controlling neuroinflammation (24). It has been reported that SalB improves neuronal damage in Parkinson's disease by inhibiting oxidizing reaction and restoring mitochondrial function (25). In depression research, preliminary evidence shows that SalB alleviates depression-like state through reducing inflammation and oxidative stress (26). It can be seen that SalB has an antidepressant-like effect. Another study found that SalB has a high binding affinity with JAK protein (binding energy is −10.3 kcal/mol), which provides a theoretical possibility for targeting this pathway (27). However, there is still a lack of systematic empirical research on whether SalB exerts antidepressant impacts via regulating JAK-STAT signaling pathway.

The chronic unpredictable mild stress (CUMS) model has been considered to be the best model to explore the pathophysiological mechanism of depression (28). Based on the above scientific background, this study hypothesizes that SalB may block neuroinflammation, protect neurons from damage and promote hippocampal neurogenesis by modulating the JAK-STAT signaling pathway, and ultimately exert antidepressant effects. To verify this hypothesis, we will systematically evaluate comprehensive regulation of SalB on depression-like behavior, JAK-STAT pathway activity, neuroinflammation, neuronal apoptosis, neurogenesis and synaptic plasticity in corticosterone (CORT)-induced HT22 hippocampal neuron cell injury model and CUMS-induced mouse depression model. The purpose of this research is to offer novel mechanism perspectives for elucidating antidepressant effect of SalB, and to provide solid experimental bases for developing natural antidepressant drugs targeting the JAK-STAT pathway.

## 2 Methods

### 2.1 Cell injury model and treatment

Mouse hippocampal neuronal cells HT22 was derived from SUNNCELL (SNL-202, Wuhan, China). HT22 cells were cultured in Dulbecco's Modified Eagle Medium (DMEM) complete medium [containing 10% fetal bovine serum (FBS) and 1% penicillin-streptomycin; SNM-002E, SUNNCELL] at 37 °C in a 5% CO_2_ cell incubator (MCO-170AICUVH-PJ, SANYO, Osaka, Japan). When the cell density was between 70 and 80%, the cells were digested with 0.25% trypsin for passage.

HT22 cells were seeded into 96-well plates overnight (12 h) and treated with 2.5, 5, 10, 15, 20, and 40 μM SalB (HY-N1362, MedChemExpress, Monmouth Junction, NJ, USA) for 24 h. Or HT22 cells were treated with 0, 25, 50, 100, 150, 200, and 250 μM CORT (HY-B1618, MedChemExpress) for 24 h. All cell experiments were performed with three independent biological replicates (*n* = 3). For each biological replicate, three technical replicates were set up, and the mean value was taken as the representative result for that biological replicate to minimize technical variability. Subsequently, 10 μl of cell counting kit (CCK)-8 solution (G4103, Servicebio, Wuhan, China) was added and incubated for 2 h. The optical density (OD) at 450 nm was detected by a microplate reader (1410101, ThermoFisher Scientific, Waltham, MA, USA), and the cell viability was calculated to determine the optimal administration conditions.

HT22 cells were randomly divided into Control group, CORT group, CORT+SalB low, medium and high dose group and CORT+20 μM SalB+JAK-STAT pathway activator RO8191 group. HT22 cells were pretreated with 10, 15 and 20 μM SalB for 1 h, followed by 20 μM RO8191 (HY-W063968, MedChemExpress) intervention for 1 h, and finally 200 μM CORT was added for 24 h.

### 2.2 Detection of apoptosis

After the intervention of HT22 cells, the cells were digested with trypsin, collected in a flow tube, and 100 μl Binding Buffer was added to re-suspend the cells. 5 μl PI and Annexin V-FITC (E-CK-A211, Elabscience, Wuhan, China) were added, gently vortexed, and incubated in dark for 15 min. Then 400 μl Binding Buffer was added to mix the samples, and the apoptosis was immediately detected on a flow cytometer (LSRFortessa, BD Biosciences, San Diego, CA, USA).

### 2.3 Detection of reactive oxygen species (ROS) content

DCFH-DA (D6471, Solarbio, Beijing, China) was diluted with serum-free medium at a final concentration of 10 μmol/L according to 1:1000. The HT22 cells after intervention were collected, added with 1 ml diluted DCFH-DA, incubated in dark at 37 °C for 20 min, and mixed up every 5 min. Finally, the cells were resuspended with 500 μl phosphate-buffered saline (PBS), and intracellular ROS levels were measured using flow cytometry.

### 2.4 CUMS depression mouse model

Specific pathogen-free (SPF) male C57BL/6J mice, 6–8 weeks old, weighing 18–22 g, purchased from Hualan Biological Vaccine Co., Ltd. (Henan, China). Before the start of the experiment, all mice were adaptively fed for 1 week in a SPF animal room with a temperature of 22 °C and a relative humidity of 50%, and a 12 h:12 h day and night alternation was performed. The feeding and use of animals were approved by Liaoning Electric Power Central Hospital. Mice could eat and drink freely.

The mice were divided into Control group, CUMS group, CUMS+20 mg/kg SalB group and CUMS+20 mg/kg SalB+20 mg/kg RO8191 group according to the random number table method, with 6 mice in each group. For drug administration, SalB was dissolved in sterile normal saline (0.9% NaCl) and administered via intraperitoneal injection. RO8191 was dissolved in sterile normal saline containing 5% dimethyl sulfoxide (DMSO) and administered via oral gavage. Control mice received an equal volume of the corresponding vehicle (sterile normal saline for SalB control, and 5% DMSO in normal saline for RO8191 control) according to the same schedule. CUMS is an animal model widely used in the study of depression. CUMS mice were subjected to a series of unpredictable mild stress stimuli, including fasting (24 h), water deprivation (24 h), tail clamping (2 cm from the tail tip for 1 min), continuous light (24 h), ice water swimming (4 °C, 5 min), hot water swimming (40 °C, 5 min), wet cage feeding (200 ml of water per cage, 24 h), tilt cage (45 °, 24 h), behavior restriction (1 h) and noise interference (30 min). Two kinds of stress stimulation were randomly applied daily, one of which was short-term stress (tail clamping, ice water swimming, hot water swimming, noise interference, and behavior restriction), and the other could be short-term stress or 24-h stress (fasting, water deprivation, continuous light, wet cage feeding, and tilt cage). Short-term stress was arranged at 9: 00–10: 00 a.m. or 15: 00–16: 00 p.m.; the 24-h stress began at 17: 00 p.m. daily and lasted until 17: 00 p.m. the next day. If the day contains a 24-h stress, the stress is initiated at 17: 00, and the other short-term stress is scheduled to be completed at 9: 00–10: 00 on the same day; if there were two kinds of short-term stress on the same day, they were arranged at 9: 00–10: 00 a.m. and 15: 00–16: 00 p.m., respectively, with at least 5 h between the two stimuli. In order to ensure the unpredictability of stress and avoid adaptation of experimental animals, the interval between two stimulations of the same stressor is greater than 5 days. The experiment lasted for 49 days (29). The mice in the Control group were not given any stimulation and maintained normal drinking water and feeding. On the 21st day of CUMS modeling, mice in the SalB group were intraperitoneally injected with 20 mg/kg SalB, mice in the RO8191 group were intragastrically administered with 20 mg/kg RO8191, and the remaining mice were intragastrically administered with the same amount of normal saline for 28 days.

The mice were first subjected to behavioral tests, and then the mice were anesthetized and decapitated. The complete brain tissue was taken out, the hippocampus was stripped, and the DG and CA1 regions were isolated. Parts of DG and CA1 regions were fixed with 4% paraformaldehyde, dehydrated with gradient ethanol, and sliced continuously using a slicer (RM2255, Leica, Wetzlar, Germany). The thickness was about 4 μm. The rest of the hippocampus was frozen at −80 °C.

### 2.5 Behavioral test

After the 49-day CUMS modeling and administration intervention, the behavioral test was performed 1 h after the last administration. In order to ensure objectivity, the operator blinded the animals and ensured that they were not within the line of sight of the mice. When changing animals, clean the site with 75% ethanol to eliminate odor interference.

Splash test (ST): used to assess self-care and self-motivation behavior. To induce grooming behavior, 2 ml 10% sucrose water was sprayed on the back fluff with a sprayer to make it wet. The total grooming time of mice within 5 min was recorded.

Tail suspension test (TST): used to assess desperate behavior. The mouse tail was fixed on a hook about 50 cm above the surface of the table with tape to keep the mouse head down. Each mouse was tested for 6 min, and the time for the mice to stop struggling and remain immobile was recorded.

Forced swimming test (FST): used to assess desperate behavior. The mice were placed in a transparent plastic cylinder (30 cm high, 19 cm in diameter), and the bottle was filled with normal temperature water (24 ± 1) °C to a height of 15 cm from the bottom. Each mouse was tested for 6 min, and the immobility time to stop struggling and keep floating was recorded.

Sucrose preference test (SPT): used to measure anhedonia. Before the formal experiment, each mouse was put into a cage alone and fasted for 12 h. Then the sugar water preference experiment was formally carried out, and a pre-weighed bottle of 1% sucrose solution and a bottle of pure water were given. The cane sugar solution bottle is placed on one side of the cage and the pure water bottle is placed on the other side. Sugar and water consumption was measured after 12 h. Sugar water preference rate = (sugar water consumption/ml)/(total water consumption/ml) × 100%.

Novel-suppressed feeding test (NSFT): used to assess anxiety-like behavior in mice. Before the test, the mice were fasted for 24 h. During the test, the ordinary mouse food particles were placed on the white paper platform in the center of the cage. Each mouse is placed in a corner of the box and allowed to explore for 5 min. At the end of the time, the mice were not fed, and the time of the first feeding of food particles was recorded as the incubation period according to 5 min. Mice sniffing food or handling food were not included in the incubation period, only mice eating food began to record.

### 2.6 Pathological staining

Nissl staining: After dewaxing and hydration of hippocampal tissue sections, Nissl staining solution (G1036, Servicebio) was stained for 3 min, 0.1% glacial acetic acid was differentiated for 1 min, xylene was transparent for 10 min, and neutral gum was used for mounting. Nissl bodies in the DG region were observed under a microscope (BX53, Olympus, Tokyo, Japan).

Immunohistochemistry: After the paraffin sections of the hippocampus were baked, xylene (247642, Sigma-Aldrich, St. Louis, MO, USA) was dewaxed and hydrated with gradient ethanol. After incubation with 3% H_2_O_2_ for 25 min in the dark, endogenous peroxidase was inhibited. The sections were placed in bovine serum albumin (BSA, 5%, V900933, Sigma-Aldrich) for 30 min. Antibodies against microtubule-associated protein 2 (MAP2, ab183830, 1:1000, Abcam, Cambridge, UK), Doublecortin (DCX, ab18723, 1:200, Abcam) and neuronal nuclei (Neuronal nuclei, NeuN, ab190565, 1:100, Abcam) were added and stored overnight at 4 °C. The next day, the first antibody was discarded, PBS was washed, and the second antibody (ab6728, 1:2000, Abcam) was added dropwise, incubated for 2 h, PBS rinsed, 3,3'-diaminobenzidine (DAB) (DA1010, Solarbio) coloration, hematoxylin nuclear re-staining. Gradient ethanol dehydration, xylene transparent after sealing. Six visual fields were chosen under the microscope to take photos and perform data statistics.

Terminal deoxynucleotidyl transferase dUTP nick end labeling (TUNEL) staining: Hippocampal tissue paraffin sections were subjected to dewaxing to water, DNase-free protease K (20 μg/ml, ST532, Beyotime, Shanghai, China) to repair antigens, and membrane rupture. The sections were added with TUNEL reaction solution (C1086, Beyotime) and incubated for 1.5 h. The nuclei were re-stained with 4',6-diamidino-2-phenylindole (DAPI) (C0060, Solarbio, Beijing, China) for 10 min. The staining was observed under a fluorescence microscope (IX73, Olympus), and the proportion of positive cells was counted by Image J software (National Institutes of Health, Bethesda, MA, USA) to statistically analyze the apoptosis of hippocampal neurons.

Immunofluorescence: For labeling proliferating cells, BrdU (B5002, Sigma-Aldrich) was dissolved in sterile normal saline (0.9% NaCl) at a concentration of 10 mg/ml. In the last 7 days of the experiment, mice received a daily intraperitoneal injection of 50 mg/kg BrdU. Control animals received an equal volume of sterile normal saline vehicle without BrdU. Following BrdU administration, mice were euthanized, and brains were fixed in 4% paraformaldehyde. The tissue was then embedded in paraffin and cut into 4 μm thick sections. Paraffin-embedded hippocampal tissue sections were prepared, and the tissue was dewaxed using xylene, followed by hydration through a series of ethanol dilutions. The sections were subjected to antigen retrieval by boiling in sodium citrate buffer (pH 6.0) for 20 min. To expose BrdU epitopes for antibody recognition, a deoxyribonucleic acid (DNA) denaturation step was performed by incubating sections in 2 N HCl for 30 min at 37 °C, followed by neutralization in 0.1 M borate buffer (pH 8.5) for 10 min at room temperature. Subsequently, after washing three times with PBS, a 5% goat serum blocking solution was applied and incubated for 30 min. After discarding the excess blocking solution, primary antibodies including ionized calcium binding adaptor molecule-1 (Iba-1, ab220815, 1:100, Abcam), bromodeoxyuridine (BrdU, ab308341, 1:2000, Abcam) and DCX (ab18723, 1:200, Abcam) were added and incubated overnight at 4 °C. The next day, sections were washed and incubated with fluorescent antibody Goat Anti-Rabbit IgG H&L (Alexa Fluor^®^ 488; ab150077, 1:1000, Abcam) for 2 h at room temperature in the dark. The DAPI staining solution (C0060, Solarbio) was used to stain the nucleus for 15 min. The sections were treated with an autofluorescence quencher for 5 min, washed with PBS, and coverslipped (30). Whole-section images of the hippocampal DG region were captured using a fluorescence microscope. For each animal, three coronal sections spanning the hippocampus. In each section, three randomly selected fields of view within the DG region (including both the suprapyramidal and infrapyramidal blades) were analyzed. The mean value of the three sections was calculated as the representative result for each animal. All image quantifications were performed by experimenters blinded to the group assignments using ImageJ software. The total numbers of Iba-1-positive, DCX-positive and BrdU-positive cells in the DG region were counted.

Golgi-Cox staining: FD Rapid GolgiStain™ Kit (PK401, FD NeuroTechnologies, North Bethesda, MD, USA) was used to perform Golgi-Cox staining in the CA1 regions. The fresh whole brain was taken out and immediately immersed in an equal volume of mixed A and B staining solution. After being immersed in dark at 4 °C for 24 h, it was replaced with fresh staining solution and continued to be immersed for 7 days. The brain tissue was taken out and fixed in C liquid at 4 °C for 3 days. The slices containing hippocampal CA1 area were cut by slicer with a thickness of 120 μm. After the slices were dried, distilled water, C liquid, and distilled water were used for 4 min gradient development, and neutral gum sealing. Under the microscope, pyramidal neurons with intact cell body and clear dendritic branches were randomly selected in the hippocampal CA1 region. The number of dendritic branches and the density of dendritic spines were analyzed by Image J software.

### 2.7 Western blot (WB)

Hippocampal tissues and HT22 cells were collected, washed twice with pre-cooled (4 °C) PBS, added with radioimmunoprecipitation assay (RIPA) reagent supplemented with protease inhibitor mixture (P0013B, Beyotime, Shanghai, China), lysed cells, incubated on ice for 30 min, centrifuged (4 °C, 12,000 *r*/min, 15 min), and the supernatant was the total protein. The protein was quantified by bicinchoninic acid (BCA) protein assay kit (P0010, Beyotime) and then boiled at high temperature. Equal amounts of protein (20 μg per lane for cell lysates and 30 μg per lane for tissue lysates) was separated using a 12% SDS-PAGE gel and transferred to activated polyvinylidene fluoride (PVDF) membranes (Millipore, Burlington, MA, USA). After blocking with 5% skim milk for 2 h, the primary antibody was incubated (4 °C, overnight). After washing three times with Tris-buffered saline with Tween-20 (TBST) (10 min each), the membranes were incubated with the secondary antibody [goat anti-rabbit IgG H&L horseradish peroxidase (HRP), ab6721, 1:2000, Abcam] under the transition condition (37 °C, 2 h). The ECL luminescent solution (HY-K1005, MedChemExpress) was used to enhance the fluorescence signal, and the gel imaging system (iBright CL1500, Invitrogen, Carlsbad, CA, USA) was used to detect protein bands. Band intensities were quantified using ImageJ software (National Institutes of Health, Bethesda, MD, USA), and glyceraldehyde-3-phosphate dehydrogenase (GAPDH) was used as an internal loading control to calculate relative protein expression levels.

The primary antibody used in this study: JAK2 (ab108596, 1:5000, Abcam), p-JAK2 (ab32101, 1:10000, Abcam), caspase3 (ab184787, 1:2000, Abcam), STAT1 (ab92506, 1:2000, Abcam), p-STAT1 (ab109461, 1:10000, Abcam), STAT3 (ab31370, 1:1000, Abcam), p-STAT3 (ab76315, 1:20000, Abcam), B-cell lymphoma 2 (Bcl-2, ab194583, 1:2000, Abcam), Cleaved-caspase3 (ab214430, 1:5000, Abcam), Iba-1 (ab178847, 1:1000, Abcam), Cyclooxygenase 2 (COX-2, ab179800, 1:5000, Abcam), Bcl-2 associated X protein (Bax, ab263897, 1:3000, Abcam), inducible nitric oxide synthase (iNOS, ab15323, 1:1000, Abcam), postsynaptic density protein 95 (PSD95, ab238135, 1:2000, Abcam), Synaptophysin (ab52636, 1:10000, Abcam), and GAPDH (ab181602, 1:10000, Abcam).

### 2.8 Detection of biochemical indicators

The HT22 cells were collected, and the extract was added for ultrasonication. The supernatant was collected after centrifugation (4 °C, 8,000 *r*/min, 10 min). Lactate dehydrogenase (LDH), glutathione peroxidase (GSH-Px), superoxide dismutase (SOD) and malondialdehyde (MDA) kits (BC0685, BC1195, BC5165, BC6410, Solarbio) were used to analyze LDH, GSH-Px, SOD and MDA contents in HT22 cells following the supplier's scheme. The OD value of each hole was detected by microplate reader.

Brain-derived neurotrophic factor (BDNF) and inflammatory factors were detected by enzyme-linked immunosorbent assay (ELISA) kit. BDNF, tumor necrosis factor-α (TNF-α), interleukin (IL)-6 and IL-1β (ELISA) kits were purchased from Elabscience (E-EL-M0203, E-EL-M3063, E-EL-M0044, E-EL-M0037, Wuhan, China). The hippocampus of mice in each group was taken and homogenized with normal saline. The supernatant was collected after centrifugation. The cell culture supernatant of each group was collected. All operations were carried out in strict accordance with the instructions of the kit. After the blocking solution was blocked, the standard and the sample to be tested were added, the enzyme-labeled antibody was added, the chromogenic solution was colored, the reaction was terminated, and the OD values were detected. The standard curve was drawn, and the contents of BDNF and inflammatory factors were calculated.

### 2.9 Molecular docking

The crystal structure of JAK2 protein (PDB ID: 7F7W) was downloaded from the Research Collaboratory for Structural Bioinformatics (RCSB) Protein Data Bank database (https://www.rcsb.org). The two-dimensional chemical structure of SalB was downloaded from the PubChem database (https://pubchem.ncbi.nlm.nih.gov), and its 3D structure was generated and optimized using the YINFO TECHNOLOGY platform (https://cloud.yinfotek.com/) to complete ligand preparation. Subsequently, Pymol 2.6.0 Software (Schrödinger Inc., New York, NY, USA) was used to remove water molecules and remove hydrogen atoms of the target protein. Import the above files into AutoDockTools-1.5.7 (Scripps Institute, La Jolla, CA, USA), and process and save them as pdbqt format files. The docking result files and docking energy were obtained by running Autodock. The lower the binding energy between the ligand and the receptor protein, the more stable the molecular binding and the higher the possibility of interaction. Pymol 2.6.0 software was used to visualize the results.

### 2.10 Statistical analysis

All data were expressed as mean ± standard deviation. Statistical analyses were performed using SPSS software (version 27.0, IBM Corp., Armonk, NY, USA) and graphs were generated using GraphPad Prism (version 9.0, GraphPad Software, San Diego, CA, USA). For cell experiments, the independent biological replicate (each consisting of three technical replicates) was considered the experimental unit (*n* = 3 per group). For animal experiments, the individual mouse was considered the experimental unit (*n* = 6 per group). All behavioral tests and image quantifications were conducted by experimenters blinded to the group assignments. Normality of data distribution was assessed using the Shapiro-Wilk test, and homogeneity of variances was evaluated using Levene's test. For comparisons between two groups, Student's two-tailed *t*-test was applied. For comparisons among three or more groups, one-way analysis of variance (ANOVA) followed by Tukey's *post hoc* test was performed. All behavioral tests and image quantifications were conducted by experimenters blinded to the group assignments. A *p* < 0.05 was considered statistically significant.

## 3 Results

### 3.1 SalB attenuated CORT-induced HT22 cell injury

**Figure 1:**
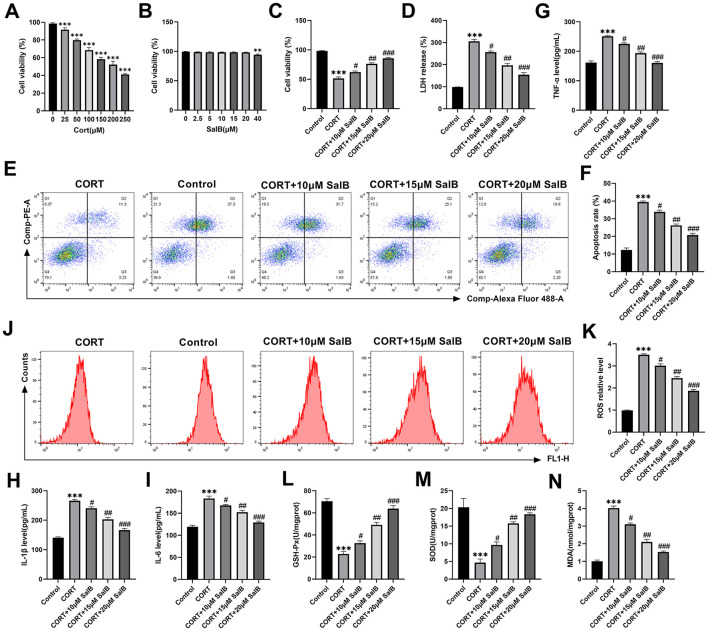
SalB attenuated CORT-induced HT22 cell damage. **(A)** HT22 cells were exposed to 0–250 μM CORT for 24 h, and CCK-8 assessed cell viability. **(B)** HT22 cells were treated with 2.5–40 μM SalB for 24 h, and CCK-8 assessed cell viability. **(C)** HT22 cells were pretreated with 10, 15 and 20 μM SalB for 1 h, and then stimulated with 200 μM CORT for 24 h. CCK-8 assessed cell viability. SalB significantly increased it. **(D)** The LDH level of the cells was detected by the kit, which was significantly reduced after SalB intervention. **(E, F)** Flow cytometry detected apoptosis level, which was significantly reduced after SalB intervention. **(G–I)** ELISA detected inflammatory factor content. SalB markedly declined TNF-α, IL-1β and IL-6 contents. **(J, K)** The intracellular ROS level was assessed using flow cytometry, which was markedly lessened after SalB intervention. **(L–N)** Oxidative stress indexes were detected by kits. SalB significantly increased GSH-Px and SOD activities and decreased MDA contents. *n* = 3, **p < 0.01, ***p < 0.001 vs. 0/Control group; ^#^*p* < 0.05, ^##^*p* < 0.01, ^###^*p* < 0.001 vs. CORT group.

The hypothalamic-pituitary-adrenal (HPA) axis is a key system for regulating stress response. CORT is a glucocorticoid whose secretion is controlled by the HPA axis and can cause neuronal damage (31). Firstly, the concentration of CORT and SalB was determined. HT22 cells were exposed to 0–250 μM CORT, and the cell viability decreased significantly with the increase of CORT dose. Among them, 200 μM CORT treatment could reduce the viability of HT22 cells to about 50% of the 0 μM CORT group (Figure 1A). Therefore, this concentration was selected to establish an *in vitro* injury model. HT22 cells were exposed to 2.5–40 μM SalB, and 40 μM SalB significantly reduced the viability of HT22 cells (Figure 1B). Therefore, 10, 15 and 20 μM SalB were selected for subsequent protection experiments. Subsequently, on this basis, after CORT induction, the cell viability of HT22 cells decreased significantly (Figure 1C), the LDH release rate increased significantly, and the apoptosis rates increased significantly (Figures 1D–F), indicating that CORT successfully induced neuronal damage. At the same time, CORT markedly enhanced inflammatory factors TNF-α, IL-1β and IL-6 contents (Figures 1G–I), and induced oxidative stress, manifested as increased ROS levels (Figures 1J, K), decreased antioxidant enzymes GSH-Px and SOD activities, and enhanced lipid peroxidation product MDA content (Figures 1L–N). SalB pretreatment reversed the above damage in a concentration-dependent manner. Specifically, SalB significantly raised viability, lessened LDH release and apoptosis rates, significantly inhibited the release of inflammatory factors, reduced ROS and MDA contents, and enhanced GSH-Px and SOD activities. Overall, SalB effectively reduced CORT-induced HT22 cell damage.

### 3.2 SalB inhibited CORT-induced activation of JAK-STAT axis in HT22 cells

**Figure 2:**
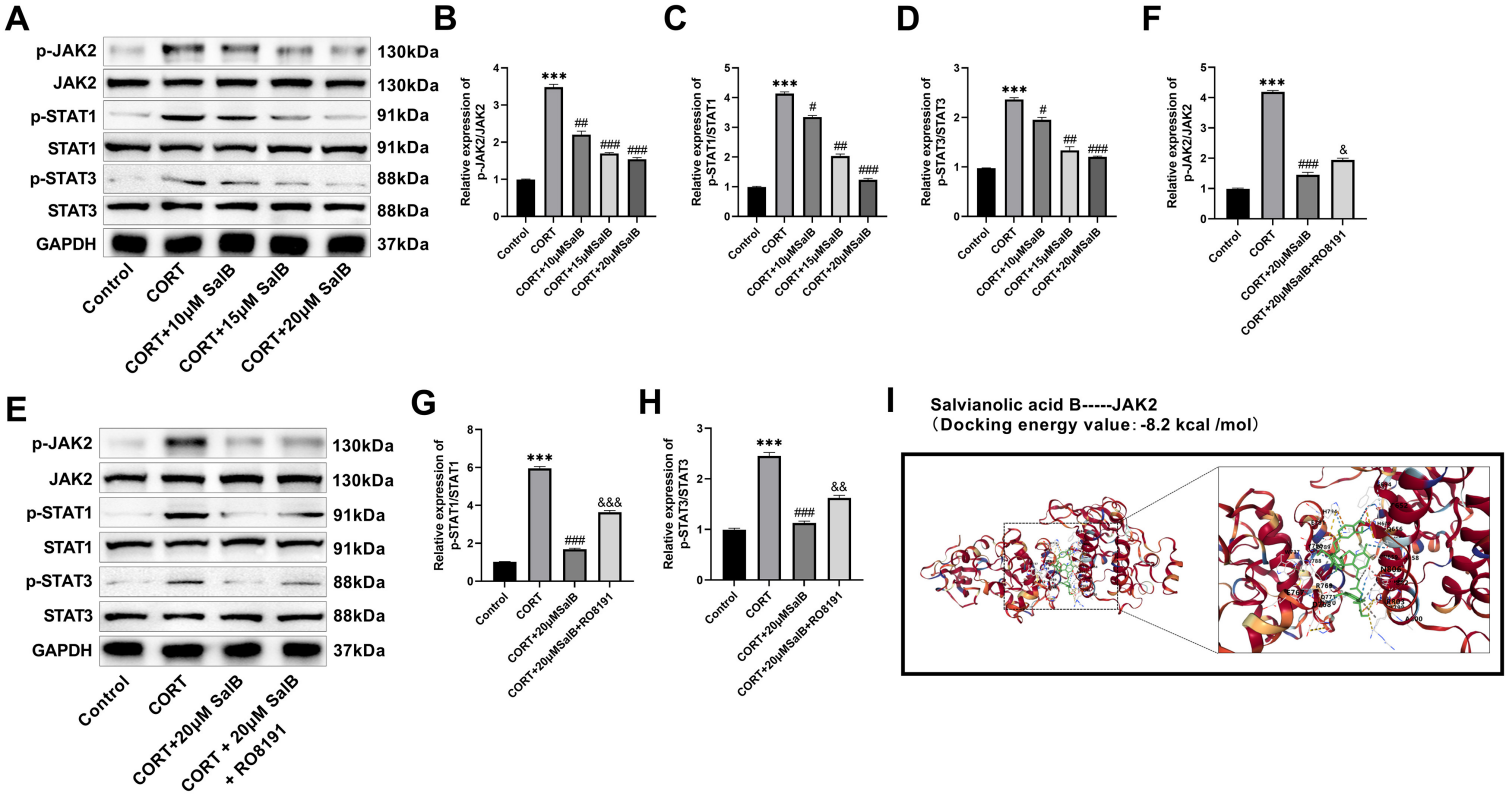
SalB inhibited the JAK-STAT pathway. **(A–D)** JAK-STAT pathway was detected using WB. SalB markedly lessened p-JAK2, p-STAT1 and p-STAT3 expressions. **(E–H)** HT22 cells were treated with JAK-STAT pathway activator RO8191, and JAK-STAT pathway was detected using WB. RO8191 partially reversed SalB's inhibitory effects on this pathway. **(I)** Molecular docking model of SalB and JAK2 protein. SalB might bind to JAK2 directly. *n* = 3, ***p < 0.001 vs. Control group; ^#^*p* < 0.05, ^##^*p* < 0.01, ^###^*p* < 0.001 vs. CORT group; ^&^*p* < 0.05, ^&&^*p* < 0.01, ^&&&^*p* < 0.001 vs. CORT+20 μM SalB group.

JAK-STAT pathway can regulate depression development. We further explored SalB's effect on JAK-STAT pathway. CORT treatment markedly up-regulated p-JAK2, p-STAT1 and p-STAT3 expressions, while the total expressions of JAK2, STAT1 and STAT3 did not change significantly. SalB pretreatment inhibited the up-regulation of the above phosphorylated proteins, and the effect of 20 μM SalB was the most significant (Figures 2A–D), suggesting that SalB effectively inhibited the activation of this signaling induced by CORT. To verify the specificity of this pathway, the study added the intervention group of JAK-STAT pathway activator RO8191. Compared with SalB intervention, JAK-STAT pathway protein expressions were significantly increased when RO8191 was added (Figures 2E–H), which partially reversed SalB's suppression of JAK-STAT pathway. In order to further predict the potential direct interaction between SalB and JAK2 protein, we performed molecular docking analysis. SalB could form stable hydrophobic interaction and hydrogen bonding with the active pocket of JAK2 protein, and its binding energy was −8.2 kcal/mol (Figure 2I), indicating that there was a strong spontaneous binding affinity between them. However, molecular docking was a computational prediction tool, and these results did not constitute direct evidence of physical binding between SalB and JAK2.

### 3.3 SalB attenuated CORT-induced HT-22 cell injury through JAK-STAT signaling pathway

**Figure 3:**
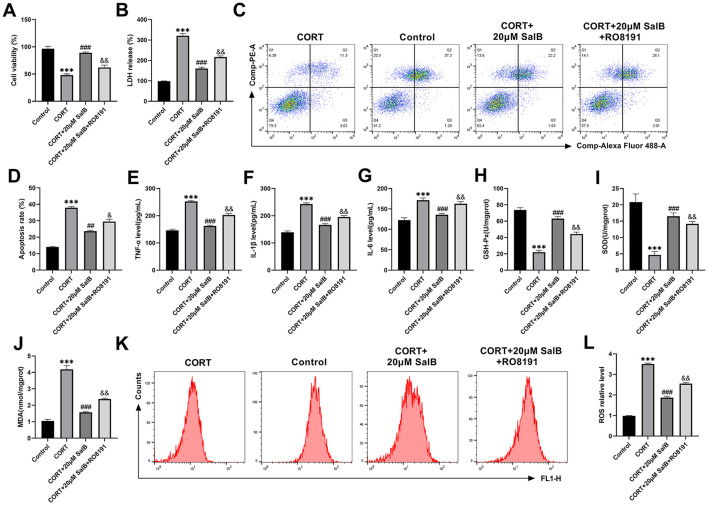
SalB attenuated CORT-induced HT-22 cell injury by JAK-STAT signaling. **(A)** CCK-8 detected cell viability. RO8191 significantly reduced cell viability. **(B)** The LDH level of cells was detected by the kit, which was significantly increased after RO8191 intervention. **(C, D)** Flow cytometry detected apoptosis level, which was significantly increased after RO8191 intervention. **(E–G)** ELISA detected inflammatory factor content. RO8191 markedly enhanced TNF-α, IL-1β and IL-6 contents. **(H–J)** Oxidative stress index was detected by kit. RO8191 significantly reduced GSH-Px and SOD activities and increased MDA contents. **(K, L)** The intracellular ROS level was detected by flow cytometry, which was significantly increased after RO8191 intervention. *n* = 3, ***p < 0.001 vs. Control group; ^##^*p* < 0.01, ^###^*p* < 0.001 vs. CORT group; ^&^*p* < 0.05, ^&&^*p* < 0.01 vs. CORT+20 μM SalB group.

Further detection showed that when RO8191 was added on the basis of SalB treatment, the cell viability of HT22 cells decreased again (Figure 3A), LDH release and apoptosis increased (Figures 3B–D), inflammatory factor contents enhanced (Figures 3E–G), ROS and MDA contents raised, GSH-Px and SOD activities decreased, and oxidative stress damage increased (Figures 3H–L). The above results clearly showed that RO8191 antagonized the protective effect of SalB on CORT-induced HT-22 cell injury. This strongly proved that the mechanism by which SalB attenuated CORT-induced HT22 cell damage were associated with inhibition of the JAK-STAT pathway.

### 3.4 SalB alleviated depression-like behaviors and improved hippocampal neuronal damage

**Figure 4:**
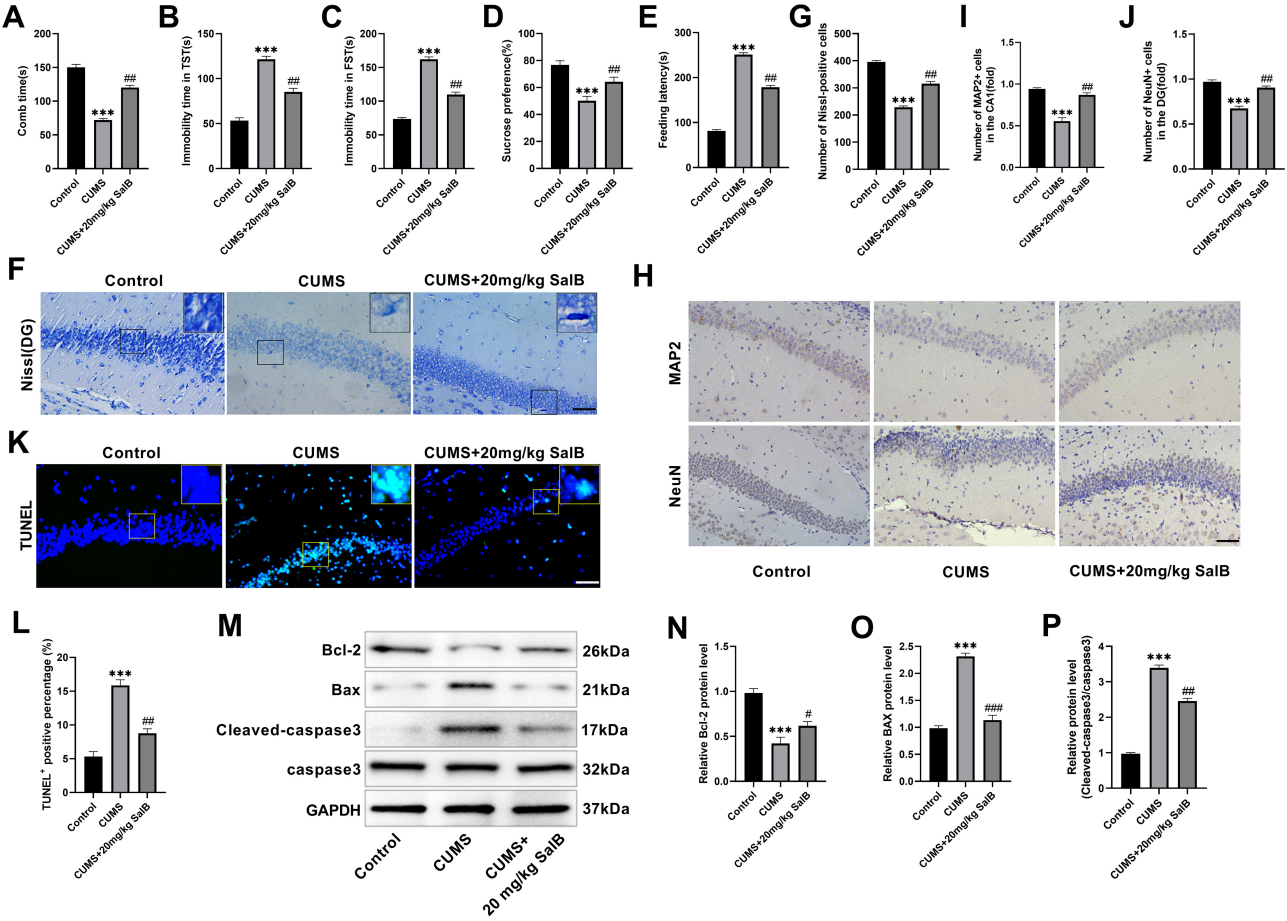
SalB alleviated depression-like behaviors and improved hippocampal neuronal damage. **(A)** CUMS was used to construct a mouse model of depression. The grooming time in ST was significantly increased after SalB intervention. **(B)** Statistics of immobility time in TST, which reduced significantly after SalB intervention. **(C)** Statistics of immobility time in FST, which reduced significantly after SalB intervention. **(D)** Statistics of sucrose preference rate in SPT, which increased significantly after SalB intervention. **(E)** Statistics of the feeding latency in NSFT, which was significantly reduced after SalB intervention. **(F, G)** Nissl bodies in DG region were detected by Nissl staining, which was significantly enhanced when SalB treatment ( × 40, 50 μm). **(H–J)** The number of MAP2 (CA1 region) and NeuN (DG region) positive neurons was determined using immunohistochemistry, which was significantly enhanced when SalB treatment ( × 40, 50 μm). **(K, L)** The apoptosis of hippocampal neurons was detected by TUNEL, which was markedly lessened when SalB treatment ( × 40, 50 μm). **(M–P)** WB detected apoptotic proteins. SalB significantly increased Bcl-2 expressions and declined Bax and Cleaved-caspase-3 expressions. *n* = 6, ***p < 0.001 vs. Control group; ^#^*p* < 0.05, ^##^*p* < 0.01, ^###^*p* < 0.001 vs. CUMS group.

The plasticity damage of the structure and function of the hippocampus is considered to be a central pathological feature of depression. CUMS was used to construct a mouse model of depression. This study next evaluated the improvement of SalB on depression-like behaviors and hippocampal neuron damages *in vivo*. Behavioral tests found that CUMS mice showed significant depression-like behaviors. The grooming time in ST was significantly reduced (Figure 4A), immobility times in TST and FST were prolonged (Figures 4B, C), sucrose preference rates in SPT were markedly lessened (Figure 4D), and the feeding latency in NSFT was prolonged (Figure 4E), indicating that CUMS mice lacked self-care motivation, increased behavioral despair, anhedonia, and increased anxiety-like behaviors. SalB intervention significantly reversed all the above behavioral abnormalities and reduced depression-like behaviors, indicating that SalB had a clear antidepressant effect at the animal level. In order to explore its neuroprotective mechanism, we analyzed the hippocampus. Nissl staining showed that Nissl body numbers of CUMS mice decreased and staining became lighter (Figures 4F, G), suggesting that neuronal metabolic activity was impaired. Immunohistochemistry showed that MAP2^+^ neurons in CA1 region and NeuN^+^ neurons in DG region of CUMS mice was significantly reduced (Figures 4H–J), indicating the loss of neuronal structural integrity and neuronal loss. Hippocampal neuronal damages are strongly associated with depression, and apoptosis represents a major way of neuronal injury (32). TUNEL staining further confirmed that apoptotic hippocampal neurons in CUMS mice was significantly increased (Figures 4K, L), and Bax and Cleaved-caspase-3 expressions were enhanced, while Bcl-2 level was reduced (Figures 4M–P). Conversely, SalB intervention effectively protected the neuronal structure, markedly raised the density of Nissl bodies, increased MAP2 and NeuN positive neurons, and reduced TUNEL positive cells and apoptosis. Overall, SalB reduced hippocampal neuronal apoptosis and protected neuronal structures and functions, thereby relieving depression-like behaviors.

### 3.5 SalB attenuated inflammation and microglial activation in hippocampal neurons from CUMS mice

**Figure 5:**
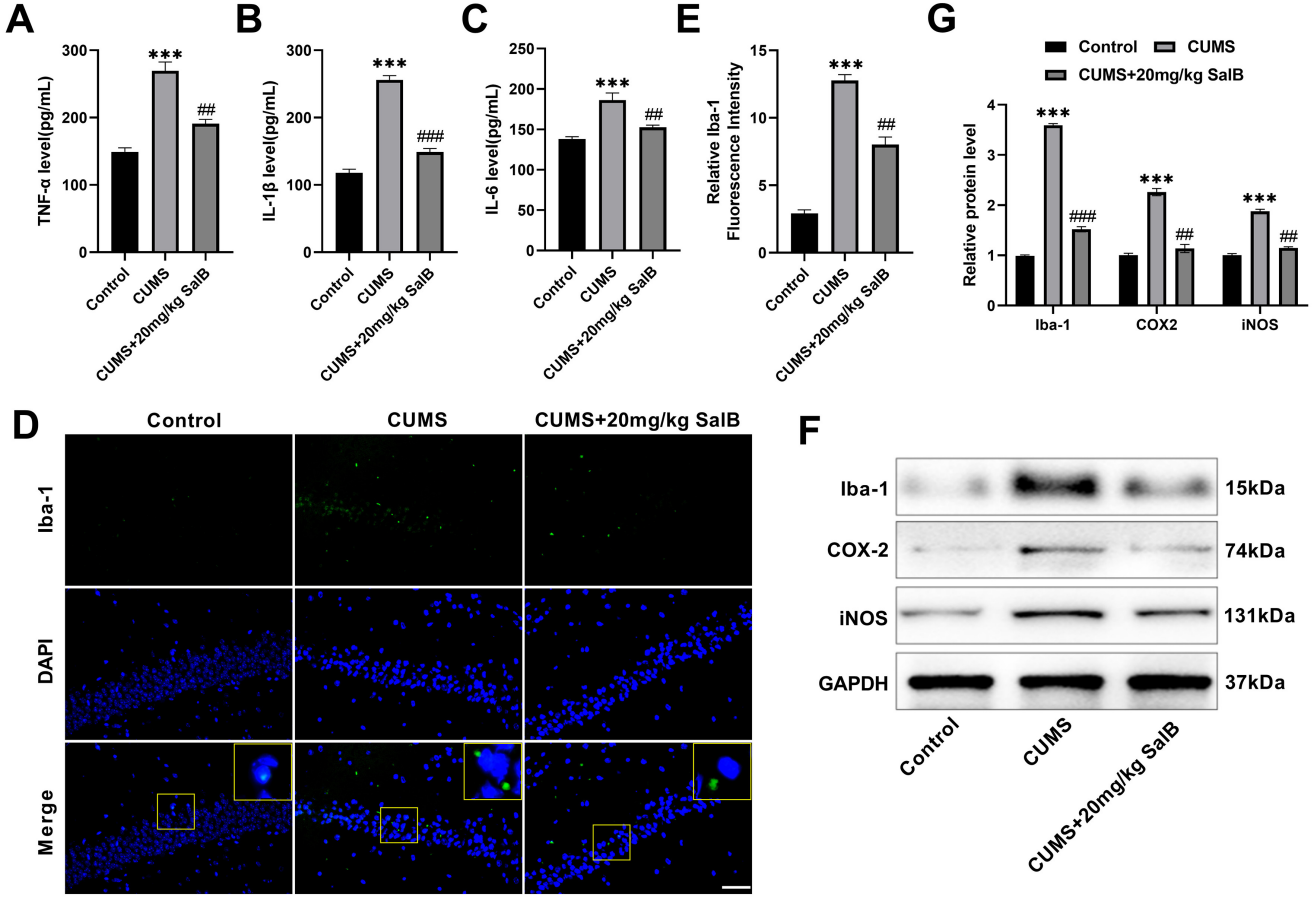
SalB attenuated inflammation and microglial activation in hippocampal neurons from CUMS mice. **(A–C)** ELISA detected inflammatory factor content in hippocampus. SalB intervention significantly reduced TNF-α, IL-1β and IL-6 contents. **(D, E)** Iba-1 fluorescence intensity in the hippocampus of mice was measured using immunofluorescence, which was significantly reduced when SalB treatment ( × 40, 50 μm). **(F, G)** WB measured Iba-1, COX-2 and iNOS proteins, which were markedly reduced after SalB intervention. *n* = 6, ***p < 0.001 vs. Control group; ^##^*p* < 0.01, ^###^*p* < 0.001 vs. CUMS group.

Excessive activation of microglia is a key link in driving neuroinflammation. The results of ELISA showed that inflammatory factor contents within hippocampus from CUMS mice were significantly increased (Figures 5A–C), indicating that CUMS successfully induced hippocampal neuroinflammation. SalB intervention significantly alleviated the degree of neuroinflammation. We further examined the activation state of microglia. Immunofluorescence staining showed that Iba-1^+^ numbers within hippocampus increased significantly from CUMS mice and decreased significantly after SalB treatment (Figures 5D, E). At the level of protein expression, the expression of microglia activation marker Iba-1, and inflammation-related enzymes COX-2 and iNOS of CUMS mice were markedly up-regulated. SalB intervention could inhibit the overexpression of Iba-1, COX-2 and iNOS (Figures 5F, G). Therefore, SalB effectively inhibited CUMS-induced hippocampal neuroinflammatory response and excessive activation of microglia.

### 3.6 SalB promoted neurogenesis in CUMS mice

**Figure 6:**
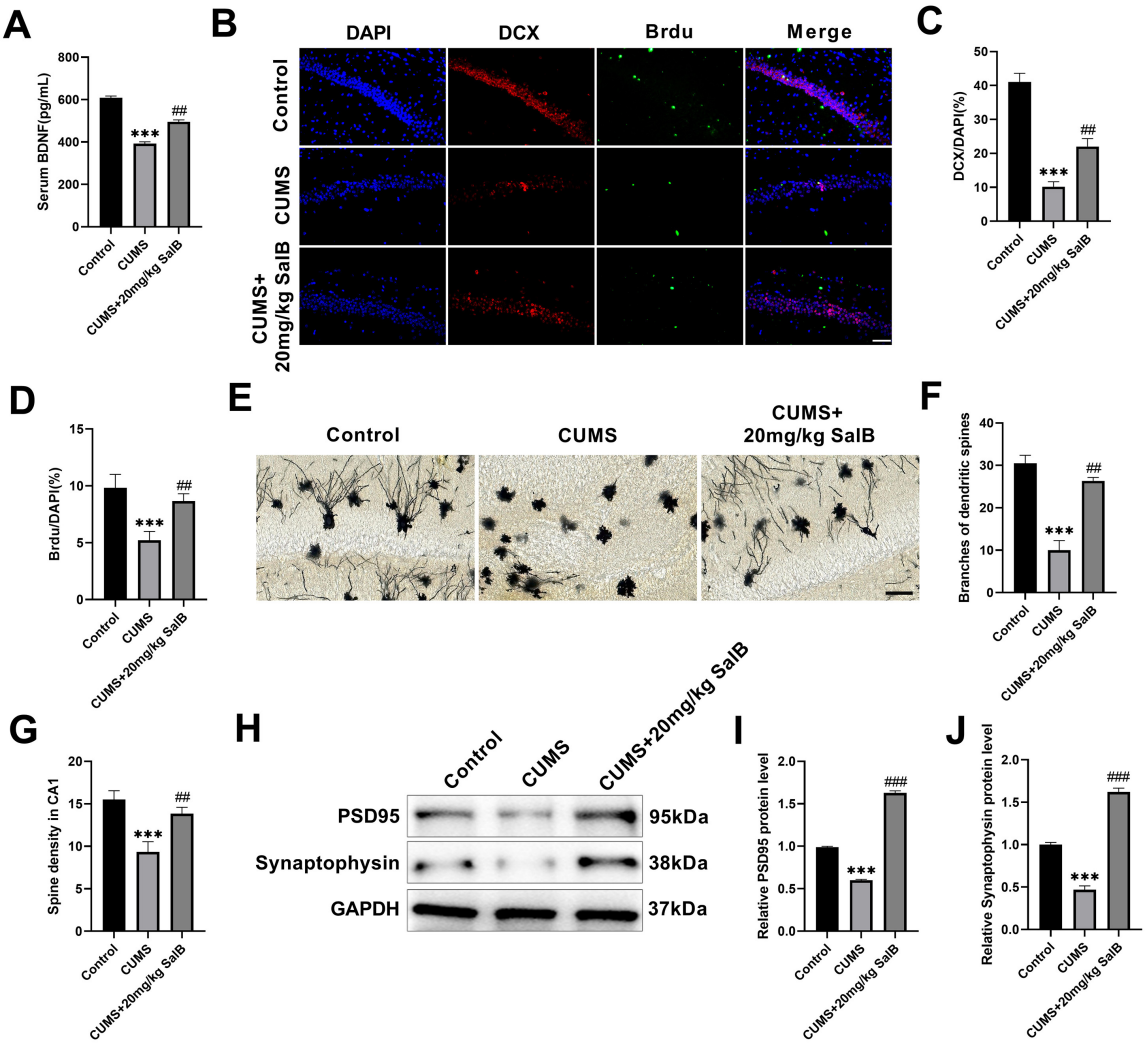
SalB promoted neurogenesis in CUMS mice. **(A)** BDNF levels in hippocampus were measured using ELISA, which were significantly raised when SalB treatment. **(B–D)** Brdu and DCX expressions in hippocampal DG region of mice were measured using immunofluorescence, which were significantly enhanced when SalB treatment ( × 40, 50 μm). **(E–G)** Golgi-Cox staining measured the number of dendritic branches and dendritic spine density, which raised significantly when SalB treatment ( × 40, 50 μm). **(H–J)** WB detected synaptic functional proteins in hippocampus of mice. SalB markedly enhanced the expressions of PSD95 and Synaptophysin. *n* = 6, ***p < 0.001 vs. Control group; ^##^*p* < 0.01, ^###^*p* < 0.001 vs. CUMS group.

Neuronal loss and synaptic damage in the hippocampus are important pathological basis of depression (33). Firstly, we detected the level of BDNF, which was closely related to neurogenesis. BDNF content within hippocampus was significantly decreased in CUMS mice and significantly increased after SalB intervention (Figure 6A). The markers of newborn neurons in DG regions were detected. Brdu and DCX positive cell numbers in CUMS mice were markedly declined, indicating that chronic stress inhibited newborn neuron proliferation and differentiation. SalB markedly raised Brdu and DCX positive cell numbers (Figures 6B–D), demonstrating its role in promoting neurogenesis. The functional output of neurogenesis depends on whether newborn neurons can be successfully integrated into existing neural networks to form functional synapses. Golgi-Cox staining showed that dendritic branches of pyramidal neurons of CUMS mice decreased, and the density of dendritic spines decreased significantly, suggesting that synaptic connection structure was damaged. SalB intervention significantly improved dendritic branching morphology and increased dendritic spine density (Figures 6E–G). WB results showed that postsynaptic marker PSD95 and presynaptic marker Synaptophysin expressions of CUMS mice was significantly down-regulated, which further confirmed the damage of synaptic function. SalB treatment significantly up-regulated the expression of PSD95 and Synaptophysin (Figures 6H–J). Therefore, SalB effectively promoted hippocampal neurogenesis in CUMS mice and improved dendritic development and synaptic integration of newborn neurons.

### 3.7 SalB alleviated CUMS-caused depression-like behaviors via inhibiting neuronal damage through JAK-STAT signaling pathway

**Figure 7:**
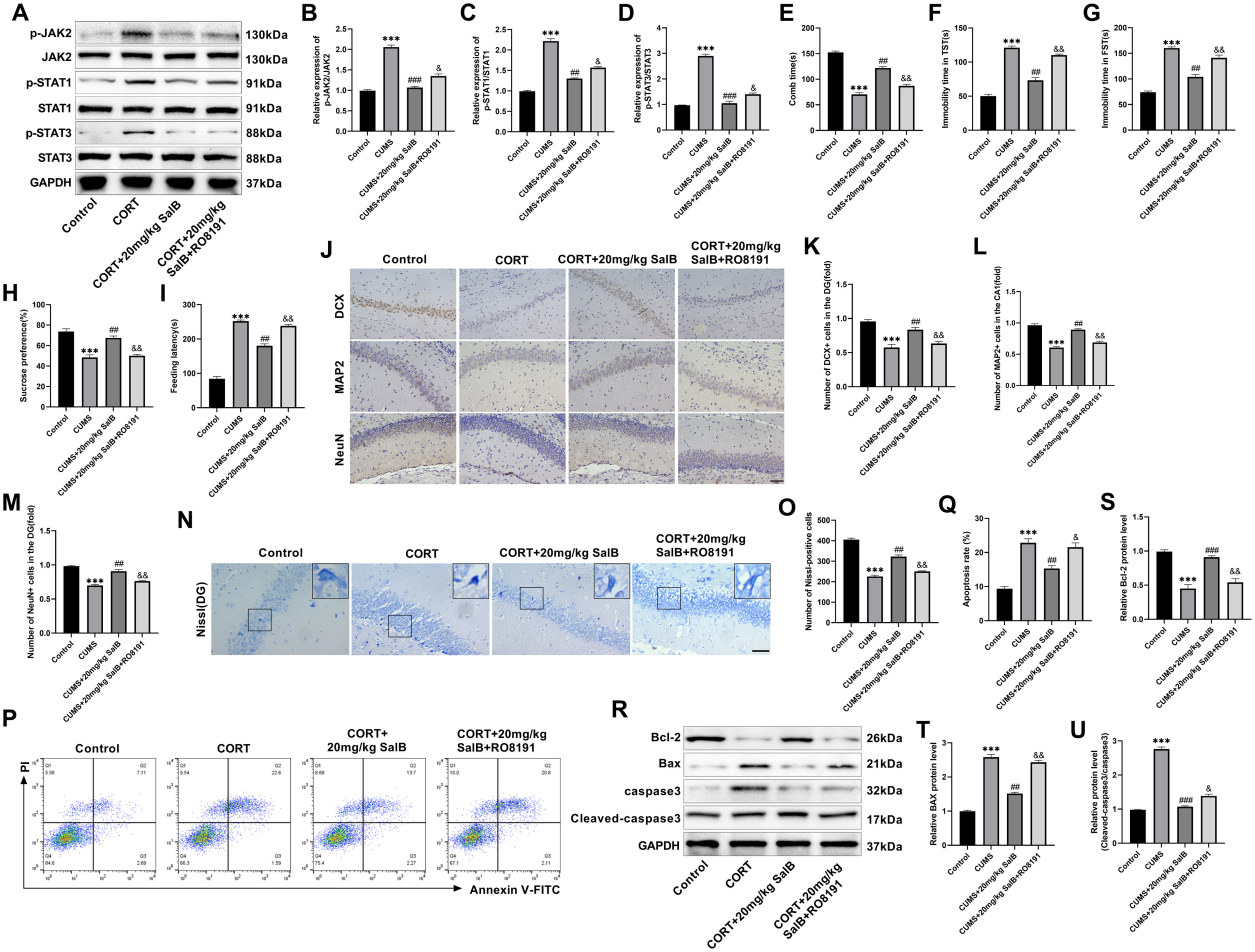
SalB alleviated depression-like behaviors via suppressing neuronal damage through JAK-STAT pathway. **(A–D)** JAK-STAT signaling was detected using WB. SalB markedly lessened p-JAK2, p-STAT1 and p-STAT3 expressions. **(E)** The grooming time in ST was counted, which was significantly reduced after RO8191 intervention. **(F)** Statistics of immobility time in TST. It increased significantly after RO8191 intervention. **(G)** Statistics of immobility time in FST. It increased significantly after RO8191 intervention. **(H)** Statistics of sucrose preference rate in SPT, which was significantly reduced after RO8191 intervention. **(I)** Statistics of the feeding latency in NSFT, which was significantly increased after RO8191 intervention. **(J–M)** DCX (DG region), MAP2 (CA1 region) and NeuN (DG region) positive neurons were detected by immunohistochemistry, they were markedly lessened when RO8191 intervention ( × 40, 50 μm). **(N, O)** The number of Nissl bodies in the DG region was detected by Nissl staining, which was significantly reduced after RO8191 intervention ( × 40, 50 μm). **(P, Q)** Cell apoptosis was examined through flow cytometry. RO8191 significantly increased hippocampal neuron apoptosis rates. **(R–U)** WB detected apoptotic proteins. RO8191 significantly decreased Bcl-2 level and increased Bax and Cleaved-caspase-3 expressions. *n* = 6, ***p < 0.001 vs. Control group; ^##^*p* < 0.01, ^###^*p* < 0.001 vs. CUMS group; ^&^*p* < 0.05, ^&&^*p* < 0.01 vs. CUMS+20 mg/kg SalB group.

Subsequently, we verified whether SalB exerted antidepressant and neuroprotective effects in a manner associated with inhibition of the JAK-STAT signaling pathway at the animal level. p-JAK2, p-STAT1 and p-STAT3 proteins of CUMS mice were markedly up-regulated, while total protein level did not change, confirming that CUMS activated JAK-STAT pathway. SalB intervention significantly inhibited the hyperphosphorylation of the pathway. When the JAK-STAT pathway activator RO8191 was given, JAK-STAT phosphorylated proteins in hippocampus of mice were significantly up-regulated (Figures 7A–D), JAK-STAT pathway was activated. Behavioral tests further showed that RO8191 markedly lessened the grooming times in ST and sucrose preference rates in SPT, prolonged the immobility times in TST and FST, and the eating latency in NSFT (Figures 7E–I), and aggravated the depression-like behavior of mice, indicating that RO8191 significantly weakened the improvement of behavioral indicators by SalB. When RO8191 treatment, DCX and NeuN positive cell numbers decreased, and MAP2 positive neurons in the CA1 region were also significantly lost (Figures 7J–M), and the Nissl bodies in the DG region were also significantly reduced (Figures 7N, O), indicating that RO8191 partially antagonized the neuroprotective effect of SalB. In addition, RO8191 also significantly enhanced the apoptosis rates of hippocampal neurons (Figures 7P, Q), up-regulated Bax and Cleaved-caspase3 levels, and decreased Bcl-2 levels (Figures 7R–U), suggesting that RO8191 blocked the anti-apoptotic effect of SalB. The above results indicated that the antidepressant, neurogenesis and neuroprotective effects of SalB were correlated with JAK-STAT signaling pathway inhibition.

## 4 Discussion

Depression clinical symptoms are mainly manifested as depression, loss of interest, cognitive impairment, accompanied by slow response, slow thinking, memory loss, inferiority, behavioral despair and even suicide. There are many animal models of depression, and different models have certain differences in behavioral performance and neurobiological mechanisms. Therefore, it is very important to compare the characteristics of neural circuits induced by different models for further understanding the pathogenesis of depression and evaluating treatment strategies (34). Among them, the CUMS model has high reliability and stability, and is the optimal model to simulate the long-term chronic low-intensity stress in humans. Its inducing factors and depression-like behaviors are highly similar to the clinical symptoms of patients with depression, and have been widely used in basic research of depression (35). In this study, ST, TST, FST, SPT and NSFT observed the depression-like behavior of depressed mice. After CUMS stimulation, the mice showed a lack of self-care motivation, increased behavioral despair, anhedonia and increased anxiety-like behavior, indicating that the depression model was successfully established, consistent with previous findings (36). SalB intervention significantly improved these depressive-like behaviors.

The primary finding is to clarify the central role of JAK-STAT pathway in the antidepressant effect of SalB. JAK-STAT signaling is vital for maintaining immune balance and tissue homeostasis (37). Inhibition of JAK-STAT signaling regulates microglia and attenuates depression-like behavior by inhibiting neuroinflammation and oxidative stress (17, 38, 39). Similar to the results, in CORT-caused HT22 cell injury models, SalB significantly suppressed the phosphorylation of JAK2, STAT1 and STAT3, accompanied by reduced inflammation, oxidizing reaction and apoptosis. Crucially, the specific pathway activator RO8191 effectively reversed all the protective effects of SalB and established a causal relationship at the cellular level. This finding is consistent with the recent research view that this pathway is a key bridge connecting chronic stress and downstream neuropathological changes (40, 41). Our results further confirmed that SalB treatment was associated with reduced JAK-STAT pathway activation.

The hippocampus is a key area related to depression (42). Chronic stress can destroy hippocampus' structure and function, and the abnormalities and lesions of the hippocampal structure seriously affect the development of depression (43, 44). Therefore, this study uses the hippocampus as a key brain area for studying depression. As an important part of the hippocampus, the DG region is a key area for regulating social behavior (45). Nervous necrosis and difficulty in regeneration in the DG region may be one of the important pathological markers of depression (46). This study confirmed the powerful neuroprotective effect of SalB from multiple levels. Pathological staining showed that SalB maintained the abundance of Nissl bodies in neurons, raised mature neurons with MAP2^+^ in CA1 regions and NeuN^+^ in DG region, and significantly reduced the apoptosis rate of neurons. Hippocampal neuronal apoptosis is considered to be an important pathological feature of chronic stress-induced depression (47), which can directly lead to a decrease in neuron numbers and dysfunction, affecting emotional regulation and cognitive function, thereby aggravating depressive symptoms (48). The commonly used molecular markers of neuronal apoptosis include Bcl-2 family proteins and Cleaved-caspase3. Xu et al. (47) found that inhibiting Bax expressions and promoting Bcl2 expressions could attenuate CUMS-induced depression-like behaviors and neuronal apoptosis. Consistent with previous studies, this study found that SalB inhibited neuronal apoptosis. RO8191 antagonized the protective effect of SalB in maintaining neuronal survival, indicating that SalB maintains neuronal homeostasis in a manner associated with inhibition of the JAK-STAT signaling pathway.

Microglia are widely associated with the physiological functions of central nervous system. Harmful stimuli can trigger abnormal activation of microglia, causing inflammatory factor production. These cytokines induce inflammatory attacks, destroy neuroplasticity, and promote neuronal damage (49). In addition to nutritional support for neurons, microglia have the function of eliminating or pruning fragile and abnormal synapses (50, 51). Inhibiting the up-regulation of inflammatory cytokines can inhibit the inflammatory response and reduce depression-like behavior (52). This study confirmed that CUMS stress significantly activated hippocampal microglia (increased Iba-1^+^ cells) and led to a surge in pro-inflammatory factors, which was consistent with the findings in various animal models (53, 54). SalB intervention effectively reversed the above changes, not only reducing the level of inflammatory factors, but also suppressing microglial activation and the expression of its effector molecules iNOS and COX-2. This effect was also antagonized by RO8191, indicating that its anti-inflammatory effect depends on the inhibition of JAK-STAT pathway. Therefore, SalB inhibits neuroinflammation through this pathway, creating a favorable microenvironment for subsequent neuronal protection and repair.

Depression is characterized by fundamental disorders of neural plasticity, including reduced neurogenesis in the hippocampus, impaired synaptic plasticity, and disorders in the stress response system (55). Enhancing neuroplasticity in the hippocampus can improve cognitive dysfunction in animals (56). Synaptic plasticity is the basic function of neural networks in response to various stimuli, and plays a role by reorganizing the structure, function and connection of neurons from molecular to behavioral levels (9). Synaptophysin and PSD95 are presynaptic and postsynaptic specific markers, respectively. High-fat diet can reduce immature neurons, synaptic density, and Synaptophysin and PSD95 expressions, thereby impairing hippocampal neuroplasticity and neurogenesis (57, 58). SalB significantly promoted the proliferation and differentiation of newborn neurons (increased Brdu^+^ and DCX^+^ cells) of CUMS mice, accompanied by up-regulation of BDNF levels. At the same time, dendrites and dendritic spines are considered to be the basis of synaptic plasticity and memory formation. SalB also improved the dendritic complexity and dendritic spine density of neurons, and up-regulated PSD95 and Synaptophysin expressions. This series of promoting effects point to the structural and functional repair of hippocampal neural circuits. It was worth noting that RO8191 also reversed the positive effects of SalB on neurogenesis and synaptophysin, which revealed this pathway negative regulation. This signaling is involved in neuronal excitability and synaptic communication changes, and targeting this pathway may reduce depression severity (16). Therefore, SalB achieves the reconstruction of hippocampal function by relieving the inhibition of neurogenesis and synaptic plasticity in a manner associated with inhibition of the JAK-STAT signaling pathway.

Of course, this study also has some limitations. First of all, although molecular docking predicted a favorable binding affinity between SalB and JAK2 (−9.6 kcal/mol), this computational result does not constitute direct evidence of physical binding. Direct interaction between SalB and JAK2 protein needs to be further verified by experimental approaches such as surface plasmon resonance (SPR) or cellular thermal shift assay (CETSA) in future studies. Secondly, the research focuses on the hippocampus, which can be extended to other emotion-related brain regions in the future to comprehensively evaluate its neural circuit effect. Third, animal experiments only used male mice and not included female animals. A large number of studies have shown that there is a significant gender dimorphism in the incidence of depression. Therefore, whether the SalB neuroprotective effect observed in this study can be extended to female animals needs to be verified by future studies using female animals or direct gender comparison. Fourthly, no positive control group (such as fluoxetine) was set in the experimental design. Although this study mainly focused on verifying the mechanism of the JAK-STAT pathway, the inclusion of established antidepressants as positive controls can provide a benchmark for evaluating the antidepressant efficacy of SalB and further verify the effectiveness of the CUMS model. Future studies should include positive control drugs to achieve a direct comparison of the efficacy of SalB with existing clinical antidepressants. Finally, the oral bioavailability of SalB is relatively low. In the future, it is necessary to optimize its pharmacokinetic characteristics with the help of new delivery systems such as nano-preparations.

## 5 Conclusion

In summary, SalB integrates anti-neuroinflammation, neuron protection and neurogenesis, thereby improving depression-like behavior, and these effects are correlated with JAK-STAT pathway inhibition. This reveals the possible molecular mechanism of SalB in the treatment of depression, and also offers solid experimental bases for developing multi-target antidepressant strategies based on natural products.

## Data Availability

The original contributions presented in the study are included in the article/supplementary material, further inquiries can be directed to the corresponding author.
